# Interplay Among Hydrogen Sulfide, Nitric Oxide, Reactive Oxygen Species, and Mitochondrial DNA Oxidative Damage

**DOI:** 10.3389/fpls.2021.701681

**Published:** 2021-08-06

**Authors:** Dandan Huang, Guangqin Jing, Lili Zhang, Changbao Chen, Shuhua Zhu

**Affiliations:** ^1^Food Safety Analysis and Test Engineering Technology Research Center of Shandong Province, College of Chemistry and Material Science, Shandong Agricultural University, Tai’an, China; ^2^College of Life Sciences, State Key Laboratory of Crop Genetics and Germplasm Enhancement, Nanjing Agricultural University, Nanjing, China

**Keywords:** hydrogen sulfide, nitric oxide, reactive oxygen species, mitochondrial DNA, damage repair pathway

## Abstract

Hydrogen sulfide (H_2_S), nitric oxide (NO), and reactive oxygen species (ROS) play essential signaling roles in cells by oxidative post-translational modification within suitable ranges of concentration. All of them contribute to the balance of redox and are involved in the DNA damage and repair pathways. However, the damage and repair pathways of mitochondrial DNA (mtDNA) are complicated, and the interactions among NO, H_2_S, ROS, and mtDNA damage are also intricate. This article summarized the current knowledge about the metabolism of H_2_S, NO, and ROS and their roles in maintaining redox balance and regulating the repair pathway of mtDNA damage in plants. The three reactive species may likely influence each other in their generation, elimination, and signaling actions, indicating a crosstalk relationship between them. In addition, NO and H_2_S are reported to be involved in epigenetic variations by participating in various cell metabolisms, including (nuclear and mitochondrial) DNA damage and repair. Nevertheless, the research on the details of NO and H_2_S in regulating DNA damage repair of plants is in its infancy, especially in mtDNA.

## Introduction

**FIGURE 1:**
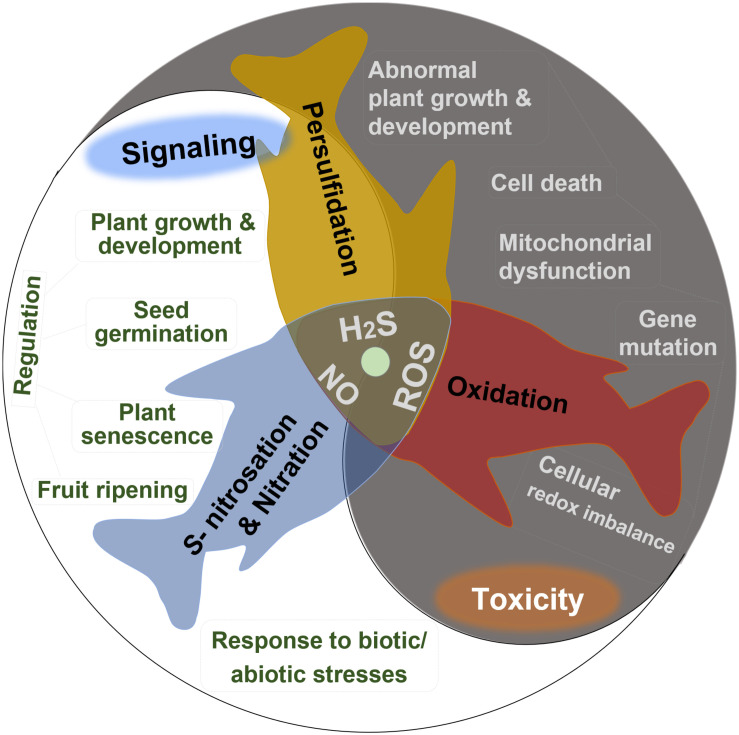
Summary of the dual bioactive role of reactive oxygen species (ROS), hydrogen sulfide (H_2_S), and nitric oxide (NO) in plants. All of them act as signaling agents by post-translational modification in many crucial physiological and biochemical processes but are toxic in excessive levels.

Hydrogen sulfide (H_2_S), nitric oxide (NO), and reactive oxygen species (ROS) including superoxide anion (O_2_⋅^–^), hydroxyl radical (HO⋅), and hydrogen peroxide (H_2_O_2_) are important intercellular signaling agents in living organisms due to their high activity, small size, and high membrane permeability (Porrini et al., 2020). For instance, H_2_O_2_ acts as an essential second messenger in the oxidative reactions with cysteine residues, producing the post-translationally modified proteins potent to redox signaling (Forman et al., 2010). H_2_S and NO perform their versatile roles in plants primarily due to the protein *S*-nitrosation and persulfidation, respectively, which are oxidative post-translational modifications of cysteine residues (Filipovic and Jovanovic, 2017; Porrini et al., 2020). NO can also react with O_2_⋅^–^ and H_2_S to produce signaling molecules peroxynitrite and *S*-nitrothiols, respectively (Hancock, 2017). In plants, all these endogenously generated reactive species appear to play multiple roles in many crucial physiological and biochemical processes (Figure 1), including modulating seed germination, maintaining plant growth and development, regulating plant senescence and fruit ripening, and improving the tolerance to biotic or abiotic stresses (Corpas, 2019b; Huang et al., 2019).

The positive effects of H_2_S, NO, and ROS depend on their proper concentrations; if not, the toxic effects show up. So, learning the exact boundary between the physiological and toxicological concentration of endogenous ROS, NO, and H_2_S contents is essential. The *in vivo* concentration of these three active substances showed great differences when measured via different methods. The entire H_2_S content ranges from 0.010 to 0.199 μmol g^–1^ FW by the methylene blue method. Using the electrode method, the H_2_S content showed a wider range, from 0.177 to 0.708 μmol g^–1^ FW (Jin et al., 2018). Low-level H_2_S and NO have acted as signaling molecules to delay the senescence of postharvest fruits and ameliorate cold stress injuries (Geng et al., 2019; Wang et al., 2021). When their concentrations reach higher levels than normal, stresses are sensed. The excessive reactive species constantly attack biomolecules, leading to severe or even irreversible oxidative modification, such as lipid peroxidation, protein oxidation, and oxidative DNA damage, which can induce membrane damage, functional changes, strand breaks, and even lead to cell death (Trachootham et al., 2008). The uncontrolled increasing level of ROS generated in plants under stress conditions often induces abnormal growth or even death of plants (Huang et al., 2019). Excessive H_2_S negatively affects the mitochondrial respiratory chain with the inhibition of cytochrome *c* oxidase by redox-reacting with the metal center (Filipovic et al., 2018).

Therefore, given the dual role depending on concentrations, maintaining the balance between production and elimination of H_2_S, NO, and ROS in plants is pivotal to continue their positive roles. The abiotic stresses elevate ROS production and may concomitantly accumulate endogenous NO and H_2_S, which can reduce oxidative stress by promoting antioxidative defenses to scavenge ROS (Bhuyan et al., 2020). Moreover, excessive ROS may convert NO to ONOO^–^, which triggers nitro-oxidative stress (Corpas and Barroso, 2013), leading to protein tyrosine nitration. H_2_S can increase the endogenous NO level (Amooaghaie et al., 2017; Corpas et al., 2019), and NO can likewise induce the accumulation of endogenous H_2_S in plants (Peng et al., 2016). Taken together, as bioactive species, ROS, NO, and H_2_S independently or collaboratively act with each other to participate in the regulation of diverse cellular processes. In this study, we summarized the production and elimination of ROS, NO, and H_2_S in plants and their roles in cellular redox balance and DNA damage and repair, attempting to describe the interplay between these reactive species from their metabolism to their regulatory performance in responding to stresses. On top of that, we described the prospects of NO, H_2_S, and ROS in the field of mitochondria and mitochondrial DNA (mtDNA) in plants.

## Metabolism of ROS, NO, and H_2_S in Plants

Generally, ROS can be produced in the cellular compartments of plants from aerobic metabolism, including respiration and photosynthesis, and regulate the life processes of plants, exhibiting its positive or toxic roles under normal or abnormal conditions (Jiao et al., 2014; Yu et al., 2017; Waszczak et al., 2018). In higher plants, ROS can be produced in chloroplasts, mitochondria, cell membranes, and peroxisomes, among which the chloroplasts and mitochondria are the main production sites and produce ROS primarily through the electron transport chain (ETC) (Fluhr, 2009). In mitochondria of plants, the ETC includes the cytochrome pathway and alternative pathway, but H_2_O_2_ or O_2_^–^ is not the end product of the reduction of oxygen in the alternate pathway (Moore and Siedow, 1991). The respiratory complexes I and III are the major sites of O_2_^⋅–^ production in mitochondria. O_2_^⋅–^ can be dismutated spontaneously or catalyzed by mitochondrial manganese-superoxide dismutase (Mn-SOD) into H_2_O_2_, which is then detoxified by peroxiredoxin (Prx), glutathione peroxidase (GPX), and ascorbate peroxidase (APX) of the ascorbate–glutathione cycle in plant mitochondria (Jimenez et al., 1997; Chew et al., 2003). H_2_O_2_ can be converted into a hydroxy radical (HO) when reacted with redox-active transition metals. Additional endogenous sources of ROS comprise the membrane-associated NAD(P)H oxidase (NOX) and xanthine oxidase (XO) (Trachootham et al., 2008). Studies showed that NOXs could regulate plant cell expansion by producing ROS, and the knockout of RHD2, an NADPH oxidase, resulted in decreased ROS content and short root hairs and stunted roots in *Arabidopsis* (Foreman et al., 2003). Besides, NADPH-mediated ROS generation was found to be related to the stomatal closing (Kwak et al., 2003), further indicating the important functions of ROS in plant growth and development. Food extracts such as hesperetin could inhibit the activity of XO, reducing oxidative stress caused by ROS (Dew et al., 2005). ROS can be scavenged via enzymatic antioxidant systems mainly including SOD, catalase (CAT), peroxidase (POD), APX, GPX, monodehydroascorbate reductase (MDHAR), dehydroascorbate reductase (DHAR), and glutathione reductase (GR), and nonenzymatic antioxidants, including NAD(P)H, glutathione (GSH), ascorbic acid (AsA), and flavonoids, which is well summarized by Huang et al. (2019).

Nitric oxide, as a small and redox-active molecule, plays versatile roles in the physiological and biochemical processes in plants (Brouquisse, 2019; Gupta et al., 2020). Dynamic monitoring of NO in the imbibed seeds showed that the amount of NO released reached a peak at 3 h; moreover, increased NO release (5 nmol min^–1^ g^–1^ DW) observed in *Arabidopsis* seeds treated with sodium nitroprusside (SNP, an NO donor) enhanced seed germination (Liu et al., 2009). For a long time, researchers dedicated themselves to clone nitric oxide synthase (NOS) from plants to figure out the biosynthetic pathway of NO but failed. Reports showed that NO can be produced from nitrite through the nonenzymatic reduction and the catalysis by nitrite reductase, nitrate reductase, or molybdoenzymes and from an oxidative route with NOS-like activity in plants (Astier et al., 2018; Kolbert et al., 2019). The mitochondrial electron transport chain (mETC) also involves the production of NO from nitrite (Alber et al., 2017; Gupta et al., 2018). Complex I (NADH: ubiquinone oxidoreductase), cooperating with rotenone-insensitive NAD(P)H dehydrogenases, regulates the production of NO under hypoxia; Complex II (succinate: ubiquinone oxidoreductase) contains Fe–S centers which can be inhibited by NO, regulating the generation of ROS; Complex III (ubiquinol: cytochrome *c* oxidoreductase) transfers an electron to nitrite to generate NO; Complex IV (cytochrome *c* oxidase) also contributes to the interconversion between nitrite and NO and the generation of ATP via the phytoglobin–NO cycle (Alber et al., 2017; Gupta et al., 2018). Complex V (F_1_F_O_-ATPase) synthesizes most of the ATP and provides energy to living cells. Tyrosine nitration caused by NO can inhibit the F_1_F_O_-ATPase activity (Nesci et al., 2017). Many studies have shown that excess NO can inhibit oxygen metabolism by the cytochrome pathway; however, the inhibition of oxygen metabolism in the alternative pathway is very weak (Millar and Day, 1996). The cDNA microarray and Northern analysis evidenced that appropriate NO levels could induce the transcription of *alternative oxidase* (*AOX*) in *Arabidopsis* cells (Huang et al., 2002). Meanwhile, the activation of NO on *AOX* transcription was also observed in tobacco (Ederli et al., 2006), and the relationship between NO and AOX was further explored by Cvetkovska and Vanlerberghe (2012) by using the transgenic material of *AOX* in tobacco, and a preliminary conclusion was drawn: AOX respiration acts to reduce the generation of ROS and reactive nitrogen species (RNS) in plant mitochondria by dampening the leak of a single electron from the ETC to O_2_ or nitrite. Excess NO may also trigger the *S*-nitrosation of complex I and other proteins.

Hydrogen sulfide has been identified as a new endogenous player in plants, and it is a mitochondrial substrate or a poison to mitochondria at low or high concentrations, respectively (Guo et al., 2016). The role of H_2_S in cells has long been a matter of controversy: Hancock and Whiteman (2014) categorize H_2_S as a referee, one for its role in responding to stresses by interacting with NO and ROS metabolism passively and another for the lack of a dedicated pathway in which H_2_S responds to such stresses. H_2_S in plants is more often considered to be a signaling molecule for its important and irreplaceable function in various physiological processes of plant cells. In plants, the diversity of sources ensured the needed supplement of H_2_S. H_2_S comes from the environment and the endogenous generation via enzymatic and nonenzymatic pathways in chloroplasts, mitochondria, and cytosols. Plants actively take up H_2_S in the atmosphere via the foliage, which can be converted into GSH in cells, leading to the increased accumulation of thiol, consequently (Ausma and De Kok, 2019). H_2_S can be generated in plants through various biosynthetic pathways, such as sulfite reductase (SiR): converting sulfite to H_2_S, cysteine desulfhydrase (CD): converting cysteine to pyruvate and H_2_S, and cysteine synthase (CS): converting L-cysteine to O-acetyl-L-serine and H_2_S. Mitochondrial β-cyanoalanine synthase (β-CAS) catalyzes the conversion of cyanide and L-cysteine to β-cyanoalanine producing H_2_S (Gotor et al., 2019). Nitrogenase Fe-S clusters also contribute to the generation of H_2_S from L-cysteine in mitochondria (Liu et al., 2015). H_2_S can be detoxicated by sulfide: quinone oxidoreductase (SQR), superoxide dismutase (SOD), or *O*-acetylserine(thiol)lyase C (OAS-TL C). SQR converts H_2_S to persulfides, which can be transferred into the conversion between GSH and glutathione persulfide (GSSH), and the sulfur is then sequentially oxidized by the mitochondrial sulfur dioxygenase ETHE1 to sulfite and by sulfite oxidase (SO) to sulfate or thiosulfate regenerated by thiosulfate sulfurtransferase (TST), transferring sulfur from GSSH to sulfite (Olson, 2018). Mitochondrial Mn-SOD also catalyzes the oxidation of H_2_S into polysulfides (D’Imprima et al., 2016; Olson, 2018). OAS-TL C could transfer sulfide to O-acetylserine to form cysteine in the mitochondria of *Arabidopsis* (Birke et al., 2015).

## H_2_S, NO, and Redox Balance in Plants

Reactive oxygen species, reactive sulfur species (RSS), and RNS burst in plants under both biotic and abiotic stress could cause the plants to responds to abnormal conditions (Jia et al., 2016; Yu et al., 2017; Corpas, 2019a; Hancock, 2019) and participate in the ripening of fruit (Muñoz-Vargas et al., 2020). During the ripening and stress response processes, ROS accumulation is ubiquitous in several sub-organelles of the cell, such as mitochondria, chloroplasts, and peroxisomes. Accompanied with this, intracellular RNS, such as NO, also increased, which alleviated oxidative damage caused by excessive accumulation of ROS via the intracellular antioxidant pathway (Zhu et al., 2014). The increasing intracellular NO could also delay the maturation and senescence of fruits by regulating ethylene biosynthesis and the ethylene signal transduction pathway and by affecting the genes of cell wall degrading enzymes (Lin et al., 2020). Mutation of SlLCD1, the H_2_S-producing enzyme, lowered the H_2_S content and accelerated fruit ripening in tomato, while exogenous H_2_S treatment in unripe fruits of tomato suppressed the expression of the ripening-related gene, suggesting that H_2_S is involved in the regulation of fruit ripening (Hu et al., 2020).

Pretreatment with exogenous H_2_S could upregulate the activities of antioxidant enzymes to remove excessive ROS and reduce oxidative damage in Chinese cabbage roots, therefore alleviating the growth inhibition caused by cadmium (Zhang et al., 2015). Exogenous H_2_S could decrease mitochondrial permeability transition and ROS contents but increase mitochondrial membrane fluidity, mitochondrial membrane potential, and antioxidative enzyme activities in roots of *Malus hupehensis* under NaCl stress (Wei et al., 2019). The salinity-induced augmentation of H_2_S and NO levels is associated with the increase of L-cysteine and L-arginine and the induction of the enzymes involved in the biosynthesis of H_2_S or NO (da Silva et al., 2017). Sodium hydrosulfide (NaHS) alleviates oxidative damage by increasing the activities of SOD, CAT, POD, and APX, promoting the transcript level of *CsNMAPK* and the accumulation of endogenous NO through the MAPK/NO signal pathway in cucumber against excess nitrate stress (Qi et al., 2019). The enhanced accumulation of GSH induced by H_2_S alters the redox of the cell, which may consequently increase the tolerance of plants to environmental stress (Noctor et al., 2012).

**FIGURE 2:**
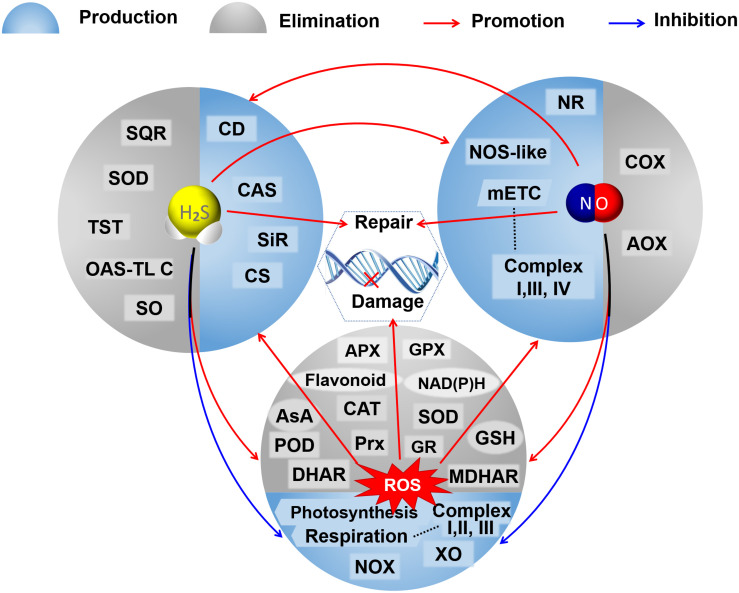
A schematic graph of the crosstalks among H_2_S, NO, and ROS in their metabolisms and regulation on DNA oxidative damage and repair. The booming ROS could trigger the accumulation of H_2_S and NO by promoting the activities of enzymes involved in their bioproduction under abnormal conditions. Both H_2_S and NO could reduce oxidative stress by inhibiting the production of ROS, promoting antioxidative defenses to scavenge excessive ROS, and mutually elevating their production. Both H_2_S and NO mediate the repair of DNA oxidative damage caused by ROS. AOX, alternative oxidase; APX, ascorbate peroxidase; AsA, ascorbic acid; CAT, catalase; CAS, cyanoalanine synthase; CD, cysteine desulfhydrase; CS, cysteine synthase; COX, cytochrome c oxidase; DHAR, dehydroascorbate reductase; GSH, glutathione; GR, glutathione reductase; GPX, glutathione peroxidase; mETC, mitochondrial electron transport chain; MDHAR, monodehydroascorbate reductase; NR, nitrate reductase; NOX, NAD(P)H oxidase; NOS-like, nitric oxide synthase like; OAS-TL C, O-acetylserine(thiol)lyase C; POD, peroxidase; Prx, peroxiredoxin; SQR, sulfide: quinone oxidoreductase; SO, sulfite oxidase; SiR, sulfite reductase; SOD, superoxide dismutase; TST, thiosulfate sulfurtransferase; XO, xanthine oxidase.

Both NaHS and SNP can increase the endogenous NO level and enhance the antioxidant enzyme activities in Bermuda grass under lead stress (Amooaghaie et al., 2017). Exogenous NaHS increases the content of endogenous H_2_S and the activity of L-cysteine desulfhydrases (L-CD) in tomatoes under nitrate stress and induces the synthesis of NO through nitrate reductase and not NOS (Liang et al., 2018). Exogenous SNP could also induce the accumulation of endogenous H_2_S by increasing the activities of L-CD, OAS-TL, and β-CAS, and exogenous NaHS enhances the NO-induced hypoxia tolerance in maize (Peng et al., 2016). However, exogenous NO decreases the activities of L-/D-CD, OAS-TL, and SiR and increases the activity of β-CAS, leading to a decrease in the contents of endogenous H_2_S, cysteine, and sulfite in peaches during cold storage (Geng et al., 2019). Treatment of exogenous H_2_S at high concentration triggered the activation of MPK6, consequently inducing the production of NO, and in turn, the exogenous H_2_S-mediated changes in auxin distribution were regulated by NO produced here, resulting in the inhibition of the primary root growth in *Arabidopsis* (Zhang P. et al., 2017). cPTIO (as an NO scavenger) and sodium tungstate (as an inhibitor of nitrate reductase) increase the H_2_S content by sustaining the activities of L-/D-CDs, OAS-TL, and SiR; L-NAME (as an inhibitor of NOS-like activity) improves the H_2_S content mainly by maintaining the D-CD activity, suggesting that there would be different interactions between the NO biosynthesis and the H_2_S metabolism (Geng et al., 2019). Also, hypotaurine (as the specific scavenger of H_2_S) reduces the endogenous H_2_S levels and reverses the responses induced by NaHS but cannot completely reverse the responses induced by SNP; while cPTIO quenches the effects of both NaHS and SNP on Pb tolerance of *Sesamum* (Amooaghaie et al., 2017). These results also suggest that there would be a two-sided link between the signal molecules of H_2_S and NO (Figure 2) and that H_2_S increases the NO production and, subsequently, NO tightly regulates feedback of the H_2_S biosynthesis (Amooaghaie et al., 2017). The complicated relationships between the H_2_S and the NO signaling cascade might depend on their respective concentrations, the different physiological and biochemical processes, different tissues and organs, and different species of plants under normal conditions or varied conditions of abiotic stress.

The current knowledge of the regulation by H_2_S, NO, and ROS on plant defense against abiotic stress has been well reviewed (Bhuyan et al., 2020). Interactions among H_2_S, NO, and ROS in mitochondria, cytoplasm, and chloroplast influenced the responses of the plant to abiotic stresses and the processes of ripening and senescence (Hancock and Whiteman, 2016; Zhang P. et al., 2017; Muñoz-Vargas et al., 2018; González-Gordo et al., 2020). To survive, plants try to maintain a proper cellular redox balance. However, the interactions among H_2_S, NO, and redox balance are still not precise and need to be further improvised.

## Mitochondrial DNA Oxidative Damage and Repair in Plants

The mitochondrion has its own DNA (mtDNA), which is highly variable in size and structure depending on the species. The mtDNA in plants is larger than human mtDNA, typically around 200–400 kb, which can be much bigger, reaching up to 11.3 Mb, and can encode about 20 additional genes in comparison to animals (Chevigny et al., 2020). The mtDNA is located near the electron transfer chain and encodes many critical proteins for the assembly and activity of the mitochondrial respiratory complexes (Alencar et al., 2019). The mtDNA is a circular molecule and is packed by proteins including prohibitins, the ATPase family AAA domain-containing protein 3 (ATAD3), mitochondrial transcription factor A (TFAM), DNA polymerase gamma, catalytic subunit (POLG), etc., forming a nucleoid that uniformly distributes within the mitochondrial matrix, which is essential for mitochondrial functions.

Proper levels of biological oxidations originating in mitochondria fulfill the beneficial roles in redox homeostasis, while excessive oxidants overwhelming the antioxidant defenses cause redox imbalance, disrupt mitochondrial function, and lead to dysfunction, aging, and cell death (Piantadosi, 2020). ROS (especially HO⋅) have a single electron and are prone to nucleophilic attack on DNA molecules, resulting in changes such as modification of DNA sequences, causing pairing and coding errors during DNA replication, and gene mutations (Poetsch, 2020). The DNA oxidative damage includes base modifications, abasic sites, and strand breaks (Gonzalez-Hunt et al., 2018). The stability of the mtDNA is essential for proper mitochondrial function. However, the mtDNA can more easily be injured by ROS on account of its proximity to the site of ROS generation and the impotent mtDNA repair (Trachootham et al., 2008). For example, exogenous H_2_O_2_ treatment resulted in the oxidation of Polγ and reduction in exonuclease activity, which in turn converted the high fidelity Polγ into an editing-deficient polymerase, leading to an increase in mtDNA mutations (Anderson et al., 2019).

The preservation of DNA integrity is necessary for ensuring unperturbed transcription (Lans et al., 2019). Unlike other cell macromolecules, damaged DNA cannot be replaced and can only maintain its integrity through direct damage reversal, mismatch repair (MMR), nucleotide excision repair (NER), base excision repair (BER), and recombination pathways [targeting DNA damages of double-strand breaks (DSBs) and single-strand gaps (SSGs)] (Chevigny et al., 2020). Direct damage reversal is the simplest way with the activities of photolyase, alkyltransferase, and dioxygenase to restore the damaged base of cellar DNA without excision of the base or the phosphodiester backbone (Yi and He, 2013). For example, photolyase could use visible light to transfer electrons from FADH^–^ to cyclobutane pyrimidine dimers (CPDs), a major UV-induced lesion in DNA in plants, resulting in CPD splitting (Zhang M. et al., 2017); alkyltransferase could repair O(6)-methylguanine in DNA by transferring the methyl group (Pegg, 2000); and dioxygenase could also demethylate DNA methylation by oxidizing 5-methylcytosine (5-meC) (Wu et al., 2018).

The non-canonical base pairing and insertion–deletion loops can be repaired via the MMR pathway, which is usually associated with the replication machinery in the nuclear matrix but is unclear in the mitochondria (Chevigny et al., 2020). The NER pathway repairs the lesions of DNA caused by UV radiation by removing DNA-binding lesions and adducts, creating a gap, which will be filled by synthesizing damage-free DNA by polymerases to finally be ligated by sealing the nick (Kobaisi et al., 2019).

However, NER and MMR pathways do not exist in plant mitochondria (Van Houten et al., 2018; Wynn et al., 2020), and BER is the primary repair pathway for mtDNA oxidative damage (Ferrando et al., 2019). The deaminations, oxidations, alkylations, and single-strand breaks of DNA can be repaired via the BER pathway (Alencar et al., 2019), the glycolytic excision of the damaged base activated the BER, and the diversity of DNA glycosylases that specifically recognize different types of lesions determined the efficiency of BER (Krokan et al., 1997). Uracil-DNA glycosylase (UNG) from *Arabidopsis* is imported into the mitochondria (Boesch et al., 2009), combined with UNG found in maize and potato (Bensen and Warner, 1987; Ferrando et al., 2019), indicating that UNG is present in the mitochondria of plants and contributes to the repair of the mtDNA. In addition, double-strand break repair (DSBR) is suggested to be a general system of repairing many DNA lesions in plant mitochondria (Wynn et al., 2020). Among the four distinct DSBR pathways, including non-homologous DNA end joining (NHEJ), alternate end joining (a-EJ), homologous recombination (HR), and single-strand annealing (SSA), HR is considered as the primary DNA repair pathway in the mitochondria of plants (Chevigny et al., 2020).

## H_2_S and NO Affect mtDNA Oxidative Damage

Oxidative damage of nuclear DNA and mtDNA can be induced by the excessive accumulation of ROS in plant cells, which could also cause epigenetic variations in plants, such as DNA methylation/demethylation (Katsuya-Gaviria et al., 2020; Nagaraja et al., 2021) and histone modifications (Zheng et al., 2021) influencing plant development and growth. DNA damage caused by ROS could trigger the nuclear redox network and affect DNA metabolism through redox-dependent regulatory mechanisms comprising redox buffering and post-translational modifications, such as the thiol-disulfide switch, glutathionylation, and *S*-nitrosation (Cimini et al., 2019). Recently, it has been found that ROS can function as catalysts of DNA methylation (Wu and Ni, 2015; Teng et al., 2018). As mentioned before, both NO and H_2_S within moderate concentration could maintain redox balance by involving the ROS metabolism and the antioxidant system, scavenging excess ROS and, thus, mitigating the oxidative DNA damage; moreover, NO and H_2_S were verified to regulate the expression and post-transcriptional modification of proteins related to DNA oxidative damage and repair (Figure 2).

Nitric oxide and H_2_S can be involved in the DNA oxidative damage via epigenetic modification. The most extensively studied and characterized epigenetic modification of DNA is the methylation of cytosine (C) with an addition of a methyl group to carbon 5 (C5) of the pyrimidine ring (5-meC) (Law and Jacobsen, 2009; Michalak et al., 2013). The methylation state of plant genomic DNA will change into the hypermethylation/hypomethylation form to affect the structure of chromatin and DNA conformation, DNA stability, and the way DNA interacts with proteins, as well as the expression of related genes under abiotic stress (Liu et al., 2017; Zhang et al., 2018). Besides, DNA demethylation, mediated by ten-eleven translocation dioxygenase (TET) 3, is reported to be crucial for efficient repair of DNA damage (Jiang et al., 2017). Studies on the roles of NO and H_2_S in DNA/mtDNA oxidative damage are more advanced in mammals. For example, mtDNA haplogroup J, which was proved to be associated with several multifactorial diseases and aging, modulates NO production (Fernández-Moreno et al., 2011), and people carrying the mtDNA haplogroup J show lower mitochondrial oxidative damage (Martínez-Redondo et al., 2010); reduction of NO and DNA/RNA oxidation products were observed in patients with systemic lupus erythematosus, and NOx levels and DNA/RNA oxidation products were inversely and independently associated (Iriyoda et al., 2017), all of these indicating that NO is associated with DNA oxidative damage. NO takes part in the regulation of DNA methylation, although likely to be genotoxic at high concentrations. Excessive NO can cause the deamination of cytosine to uracil in single-stranded DNA cytosine residues, resulting in DNA/mtDNA damage, histone deamination (Merchant et al., 1996). As an NO donor, SNP at high concentration inhibits the growth of rice seedlings, which is associated with hypomethylation at the CHG sites (H=A, C, or T) of genomic DNA and the transcriptional activation of genes and transposable elements, and the DNA methylation caused by SNP is inherited by the next generation (Ou et al., 2015). Proper concentration of exogenous NO mitigates the increase of genomic template instability, DNA methylation, and retrotransposon polymorphism caused by copper stress by increasing the efficiency of the antioxidative system in lettuce (Yagci et al., 2019). Studies in smooth muscle cells and aorta tissues of mice found that a sufficient level of H_2_S was able to inhibit TFAM promoter methylation and maintain the mtDNA copy number (Ou et al., 2015). Methyl in trans-methylation reactions are tightly coupled with the activated methyl cycle, a crucial contributor to DNA and RNA methylation in stress-exposed plants (Rahikainen et al., 2018). As a donor of the methyl in trans-methylation reactions, *S*-adenosyl-l-methionine (SAM) was associated with the production of H_2_S (Eto and Kimura, 2002). NO can regulate enzymes such as S-adenosylhomocysteine hydrolase/homologous gene silencing 1, methionine synthase, and S-adenosyl methionine synthase/methionine adenosyltransferases in SAM synthesis via *S*-nitrosation and tyrosine nitration (Lindermayr et al., 2005; Kumar et al., 2020), indicating the crosstalk between H_2_S and NO in regulating DNA methylation/demethylation.

On the other hand, NO and H_2_S could participate in the DNA/mtDNA damage (repair) via post-transcriptional modification of proteins. NO-mediated increase in DNA-dependent protein kinase catalytic subunit (DNA-PKcs), a key double-strand DNA break repair enzyme involved in non-homologous end-joining, demonstrated the presence of a new and highly effective NO-mediated mechanism for DNA repair through *S*-nitrosation and transcriptional regulation (Xu et al., 2000). NO also modifies histone methylation by regulating protein arginine methyltransferase activity by *S*-nitrosation, upregulating the expression of the gene encoding lysine methyltransferase, which is the predominant mechanism for transduction of NO bioactivity (Hussain et al., 2016; Blanc and Richard, 2017). Similarly, the NO donor treatment resulted in tyrosine nitration and inhibition of its activity possibly through *S*-nitrosation, which involved DNA repair (Jones et al., 2009). The redox modifications, such as the *S*-nitrosation caused by NO, may inhibit histone deacetylases (HDAC 2C and 2B) and modulate histone acetylation in *Arabidopsis* (Chaki et al., 2015; Mengel et al., 2017). Histones, acetyltransferases, and methyltransferases are the targets for persulfidation (Aroca et al., 2018), suggesting that H_2_S can also participate in DNA repair like NO. Exogenous SNP and *S*-nitrosoglutathione (GSNO), as NO donors, cause the *S*-nitrosation of Cys49 and Cys53, promoting a conformational change in the secondary structure in proteins of the *AtMYB30* transcription factor and inhibiting the DNA binding ability of R2R3-MYB2 from *Arabidopsis* (Serpa et al., 2007; Tavares et al., 2014). Histone deamination could be repaired through the BER pathway, which is responsible for the repair of damaged single bases resulting from deamination, alkylation, and oxidized bases (Cimini et al., 2019). H_2_S can modify the thiol group of cysteine (-SH) in proteins into a persulfide group (-SSH) through the process of *S*-sulfhydration, which is considered as the protective mechanism for proteins against oxidative damage (Aroca et al., 2018). Cysteine residues of proteins can be modified through both *S*-nitrosation by NO and *S*-sulfhydration by H_2_S, suggesting that cysteine may be a hub between the physiological effects of H_2_S and NO, and the *S*-nitrosation and *S*-sulfhydration of cysteine may be interconvertible.

Moreover, studies in rats showed that pretreatment of NaHS attenuated Hcy-induced mitochondrial toxicity caused by excessive ROS and mito-ROS and restored ATP production and mtDNA copy numbers as well as oxygen consumption in the osteoblast (Zhai et al., 2019). NaHS significantly reduced oxidative stress and attenuated the mitochondrial damage induced by methylmercury (MeHg), and they increased DNA and RNA content in the rat cerebral cortex (Han et al., 2017), indicating the potentially protective effects of H_2_S against mitochondrial toxicity related to ROS (Zhai et al., 2019). Because of the importance of mitochondria in cells, the mechanisms of the regulation by NO and H_2_S on mtDNA oxidative damage in plants under normal or different stresses are intriguing aspects and still need to be deeply studied. However, the current studies about the regulation by NO and H_2_S on mtDNA oxidative damage in plants are still in their infancy, and there is still much research to be done.

## Conclusion and Prospects

There are crosstalks among H_2_S, NO, and ROS in the biosynthesis and physiological effects. Both H_2_S and NO can regulate ROS metabolism to maintain redox balance in plants. The redox imbalance causes DNA damage, which in turn exacerbates the imbalance in plants under normal and stress conditions. H_2_S and NO are suggested to protect DNA against damage by indirectly scavenging or removing excessive ROS or by directly modifying the components and improving the ability of the DNA repair pathway. H_2_S, NO, and ROS have many variants and can transform easily and quickly, which brings great difficulties in studying the details and even their inhibitors and scavengers regulating redox balance. At present, a great number of studies focus on the roles of NO in DNA damage repair, but the details and mechanisms that NO regulates damage repair are not clear. Compared with NO, the research on the role of H_2_S in DNA damage repair is in its infancy in plants. The damage and repair pathways of mtDNA are complicated, and the interplays among NO, H_2_S, ROS, and mtDNA damage are also intricate. In what way and with which repair pathways do H_2_S and NO regulate mtDNA oxidative damage in plants under normal or different stresses? Additionally, studies on whether the similar regulation of DNA damage repair by NO and H_2_S present in other organelles, i.e., chloroplasts possessing their DNA (cpDNA) would also be meaningful. In recent years, the development of sequencing technology (including high throughput sequencing and single-cell sequencing) offers a fast and cost-effective method for sequencing the whole mtDNA genome (Grabherr et al., 2011; Sloan, 2013). The exploitation of universal and conserved mitochondrial primers (Duminil, 2014; Pereira et al., 2018), combined with opportunities offered by the availability of complete mtDNA sequence in plant species, facilitate the mtDNA-based molecular studies. Moreover, the emergence of mitochondrial genome editing technology (RNA-free DddA-derived cytosine base editors and mitoTALENs) enables the study of mitochondrial gene functions to be carried out in-depth (Kazama et al., 2019; Arimura et al., 2020; Mok et al., 2020). Technological developments may provide the details of mtDNA damage and the roles of NO, H_2_S, and ROS in regulating the repair pathways of mtDNA damage in response to stress in plants as well. More advanced instruments and analytical methods are also needed to study the temporal and spatial changes of NO, H_2_S, and ROS in plants, but there is still a long way to go.

## Author Contributions

DH and GJ collected the references and completed the first draft. LZ and CC revised the manuscript. SZ designed the framework and edited the manuscript. All authors contributed to the article and approved the submitted version.

## Conflict of Interest

The authors declare that the research was conducted in the absence of any commercial or financial relationships that could be construed as a potential conflict of interest.

## Publisher’s Note

All claims expressed in this article are solely those of the authors and do not necessarily represent those of their affiliated organizations, or those of the publisher, the editors and the reviewers. Any product that may be evaluated in this article, or claim that may be made by its manufacturer, is not guaranteed or endorsed by the publisher.
